# Circulating microRNAs associated with prediabetes and geographic location in Latinos

**DOI:** 10.1007/s13410-020-00917-1

**Published:** 2021-02-11

**Authors:** Elena Flowers, Juan-Daniel Ramírez-Mares, Marion Velazquez-Villafaña, Ruben Rangel-Salazar, Anatol Sucher, Alka M. Kanaya, Bradley E. Aouizerat, Maria Luisa Lazo de la Vega Monroy

**Affiliations:** 1 Department of Physiological Nursing, University of California, San Francisco, 2 Koret Way, #605L, San Francisco, CA 94143-0610, USA; 2 Institute for Human Genetics, University of California, San Francisco, San Francisco, USA; 3 Medical Sciences Department, Health Sciences Division, University of Guanajuato, Guanajuato, Mexico; 4 University of California, San Francisco, San Francisco, USA; 5 Department of Medicine, University of California, San Francisco, San Francisco, USA; 6 Department of Epidemiology and Biostatistics, University of California, San Francisco, San Francisco, USA; 7 College of Dentistry, Bluestone Center for Clinical Research, New York University, New York, USA

**Keywords:** microRNA, Diabetes, Fasting blood glucose, Biomarker

## Abstract

**Background:**

Globally, type 2 diabetes is highly prevalent in individuals of Latino ancestry. The reasons underlying this high prevalence are not well understood, but both genetic and lifestyle factors are contributors. Circulating microRNAs are readily detectable in blood and are promising biomarkers to characterize biological responses (i.e., changes in gene expression) to lifestyle factors. Prior studies identified relationships between circulating microRNAs and risk for type 2 diabetes, but Latinos have largely been under-represented in these study samples.

**Aims/hypothesis:**

The aim of this study was to assess for differences in expression levels of three candidate microRNAs (miR-126, miR-146, miR-15) between individuals who had prediabetes compared to normal glycemic status and between individuals who self-identified with Latino ancestry in the United States (US) and native Mexicans living in or near Leon, Mexico.

**Methods:**

This was a cross-sectional study that included 45 Mexicans and 21 Latino participants from the US. Prediabetes was defined as fasting glucose 100–125 mg/dL or 2-h post-glucose challenge between 140 and 199 mg/dL. Expression levels of microRNAs from plasma were measured by qPCR. Linear and logistic regression models were used to assess relationships between individual microRNAs and glycemic status or geographic site.

**Results:**

None of the three microRNAs was associated with risk for type 2 diabetes. MiR-146a and miR-15 were significantly lower in the study sample from Mexico compared to the US. There was a significant interaction between miR-146a and BMI associated with fasting blood glucose.

**Conclusions/interpretation:**

This study did not replicate in Latinos prior observations from other racial groups of associations between miR-126, miR-146a, and miR-15 and risk for type 2 diabetes. Future studies should consider other microRNAs related to different biological pathways as possible biomarkers for type 2 diabetes in Latinos.

## Introduction

Type 2 diabetes is highly prevalent in individuals of Latino ancestry in both native countries of origin and in immigrants to other countries. The prevalence of type 2 diabetes in Latinos living in the United States (US) is 12.7% [1] and the prevalence of type 2 diabetes in Mexico is 14.8% [2]. Progression to type 2 diabetes occurs on a continuum, and even in the prediabetes state, harmful complications begin to occur [3]. Genetic risk factors for type 2 diabetes are common in some individuals of Latino ancestry [4, 5]. However, Latinos are characterized by highly heterogeneous genetic admixture [6], and genetic risk for type 2 diabetes between individuals who identify as Latino may vary considerably by geographic ancestry. Furthermore, environmental, social, and lifestyle factors are also important contributors to risk for type 2 diabetes [7]. The complex etiology of type 2 diabetes makes it hard to accurately identify which individuals are at greatest risk and the specific mechanisms underlying risk for a given individual or population.

MicroRNAs are short (i.e., 18–26 nucleotide) regulatory elements of translation of messenger RNAs to amino acids. Circulating microRNAs found in serum and plasma are easily measured in blood and are potential biomarkers for risk for development of type 2 diabetes, characterizing changes in expression levels prior to the onset of prediabetes or type 2 diabetes [8, 9]. Because microRNAs capture both underlying genetic risk as well as responses to environmental, social, and lifestyle factors [10, 11], they may be useful as biomarkers in two ways. The first is improved identification of which individuals are at greatest risk for type 2 diabetes. The second is information about specific patterns of gene expression in individuals at risk for type 2 diabetes, which is of particular interest for this complex condition because gene expression is driven by interactions between underlying genetic predisposition and environmental and lifestyle factors.

Prior studies on microRNAs associated with risk for type 2 diabetes have primarily been focused on non-Hispanic white and Asian populations [12]. The purpose of this study was to assess relationships between circulating microRNAs and prediabetes in individuals of Latino ancestry. We selected three microRNAs (i.e., miR-126, miR-146a, miR-15) previously shown to be associated with risk for type 2 diabetes in other racial groups to assess in this study [13, 14]. We evaluated differences in microRNA expression levels between individuals who self-identified with Latino ancestry in the United States (US) and native Mexicans living in or near Leon, Mexico, and individuals who were prediabetic compared to normal glycemic status.

## Research design and methods

### Recruitment

This was a multi-center observational cross-sectional study carried out at two different research institutions in Mexico and the US.

#### Mexico

Participants of Mexican ancestry were recruited from primary care health centers that serve the general population from the city of Leon in Guanajuato, Mexico. Participants included were between 35 and 65 years old without a previous diagnosis of prediabetes. Participants presenting with fasting glucose of 100–125 mg/dL or 2-h glucose between 140 and 199 mg/dL after an oral glucose tolerance test (OGTT) were categorized as having prediabetes (*n* = 36). Participants with fasting glucose < 100 mg/dL and 2-h OGTT < 140 mg/dL were categorized as having normal glucose tolerance (*n* = 30). Exclusion criteria included history of diabetes, hypertension, thyroid, hepatic, immune, neoplastic, or endocrine disorder; statin, glucocorticoid, or anticonvulsant use; current smoking; and > 2 drinks of alcohol/day.

#### United States

The US study sample included participants from the previously completed Practicing Restorative Yoga Metabolic Syndrome (PRYSMS) study (clinicaltrials.gov identifier NCT01024816), which tested the effects of restorative yoga versus active stretching on blood glucose in adults at risk for type 2 diabetes. Participants in the PRYSMS study were recruited from the San Francisco and San Diego areas and met the International Diabetes Federation criteria for metabolic syndrome [15]. The subset of participants who self-identified as Latino were included in this study (*n* = 21). Exclusion criteria included fasting glucose ≥ 126 mg/dL, HbA1c ≥ 7.0%, fasting triglycerides ≥ 300 mg/dL, weight ≥ 400 lbs, chronic disease, and neurological conditions that limited mobility, hospitalization for coronary heart disease within the past 6 months, current pregnancy or lactation, history of bariatric surgery, substance abuse, and use of medications affecting metabolic factors. The PRYSMS trial and the study described in this paper were approved by the Institutional Review Board at the University of California, San Francisco. All participants provided informed consent to participate in the study.

### Data Collection

#### Mexico

The enrollment visit at the Mexico site included a brief medical history and family history for diabetes using the American Diabetes Association criteria [16]. Physical activity was assessed using the Spanish version of the self-administered short International Physical Activity Questionnaire (IPAQ) forms [17]. Participant height and weight were measured with a standardized stadiometer (Seca) and scale (Tanita BC-536). Hip and waist girth were measured with an anthropometric tape (Lufkin), and body composition was assessed using a bioimpedance device (InBody). Blood glucose and lipid levels were measured from serum samples by colorimetric enzymatic assays (Spinreact). Insulin was measured from serum using an ELISA kit (ALPCO) as per the manufacturer’s instructions. Hemoglobin A1c (HbA1c) was analyzed via chromatography with Labona Check equipment and reagents in plasma. Plasma was stored at − 80 °C at both locations until used.

#### United States

The full clinical data collection protocol for the PRYSMS trial has been reported previously [18]. Participant weight was measured on a standard balance beam scale and height using a stadiometer. Waist circumference was measured using a Gullick II tape spring-tension measure at the site of maximum circumference midway between the lower ribs and the anterior superior iliac spine. The mean of two waist circumference measurements was calculated. Blood glucose was measured using an automated analyzer with an immobilized enzyme biosensor (YSI 2300 STAT Plus, YSI Life Sciences, Yellow Sprints, OH). Total cholesterol, triglycerides, and HDL-cholesterol were measured by enzymatic colorimetric methods (Quest Diagnostics, San Jose, CA), and LDL-cholesterol was calculated using the Friedewald equation [19]. Blood used for banking of plasma was collected by venipuncture. Blood was collected into vacutainers containing the preservative EDTA, centrifuged at 4 °C to separate plasma from cellular blood components, and stored at − 80 °C.

### MicroRNA quantitation

Study personnel from the Mexico site were trained in the isolation and quantitation of microRNAs at the US site. Both sites employed the same study protocol for all assays. RNA was extracted from 200 μL of plasma using the miRNeasy serum/plasma kit (Qiagen). Purified RNA was converted to cDNA using the miScript II RT Kit (Qiagen) in 20 μL reaction volumes using the miScript HiSpec buffer. Real-time quantitative polymerase chain reaction (qPCR) was used to assess relative expression of candidate microRNAs using the miScript kit (Qiagen). Experiments were carried out using a 384-well (US) or 96-well (Mexico) plate format on a Bio-Rad CFX real-time PCR machine using the manufacturer’s recommended cycling conditions. A standard curve was constructed for each microRNA target using a series of five serial dilutions. Both sites obtained at least three replicates measures per sample for each microRNA target. MicroRNA expression levels were normalized using cel-miR-39 and the global geometric mean signal of all reliably detected microRNAs [20, 21], and relative expression levels were calculated using the ΔΔCt method [22].

### Statistical analysis

Descriptive statistics and Student’s *t* test were used to evaluate demographic and clinical characteristics of participants between study sites and glycemic status (Stata version 13, College Station, TX). Pearson’s correlation coefficients were used to determine relationships between fasting blood glucose and covariates that are continuous variables. Logistic regression models were used to determine whether individual microRNAs were associated with prediabetes compared to normal glucose tolerance. Logistic regression models were also used to determine whether individual microRNAs were associated with the study site with the US as the reference site. Linear regression models were used to determine whether individual microRNAs were associated with fasting blood glucose. For all regression models, unadjusted models were first created. Next, variables that were significantly associated with prediabetes or study site were included as covariates in adjusted models. Finally, we included interaction terms for covariates that were significantly associated with individual microRNAs.

## Results

A total of 45 participants were enrolled in Leon, Mexico, and 21 participants from the US-based PRYSMS trial who self-identified as Latino were included in the study. Participants from Mexico were younger (46 ± 8 years versus 51 ± 7 years, *p* < 0.05) and had lower BMI (29.8 ± 3.8 kg/m^2^ versus 35.9 ± 8.1 kg/m^2^, *p* < 0.05) and weight (81.8 ± 11.4 kg versus 92.3 ± 18.2 kg, *p* < 0.05) (Table 1). While there were no differences in fasting blood glucose, hemoglobin A1c was higher in Latinos from the US compared to Mexicans from Mexico (6.0 ± 0.3% versus 4.4 ± 0.5%, *p* < 0.001) (Table 1). In a multivariate-adjusted logistic regression model, BMI but not age or sex was significantly lower in individuals from Mexico compared to the US (OR 0.82 (95% CI 0.71, 0.94)).

In the full study sample, 60% (*n* = 36) of participants had prediabetes compared to normal glucose tolerance (Table 2). There was no difference in the proportion with prediabetes by study site. A higher proportion of individuals with prediabetes were female (83% (*n* = 30) versus 57% (*n* = 17), *p* < 0.05) and had higher BMI (34.4 ± 6.0 kg/m^2^ versus 29.7 ± 5.8 kg/m^2^, *p* < 0.05). Fasting blood glucose was higher in individuals with prediabetes (109 ± 8 mg/dL versus 89 ± 8 mg/dL, *p* < 0.05) but there were no differences in hemoglobin A1c. In a multivariate-adjusted logistic regression model, BMI, but not age or sex, was significantly associated with risk for prediabetes (OR 1.12 (95% CI 1.00, 1.26)).

All three microRNAs were strongly significantly correlated with each other (Table 3). MiR-146a was significantly associated with BMI (*r*^2^ = 0.28, *p* < 0.05). There were no other significant correlations between individual microRNAs and age, sex, or BMI.

**Fig. 1:**
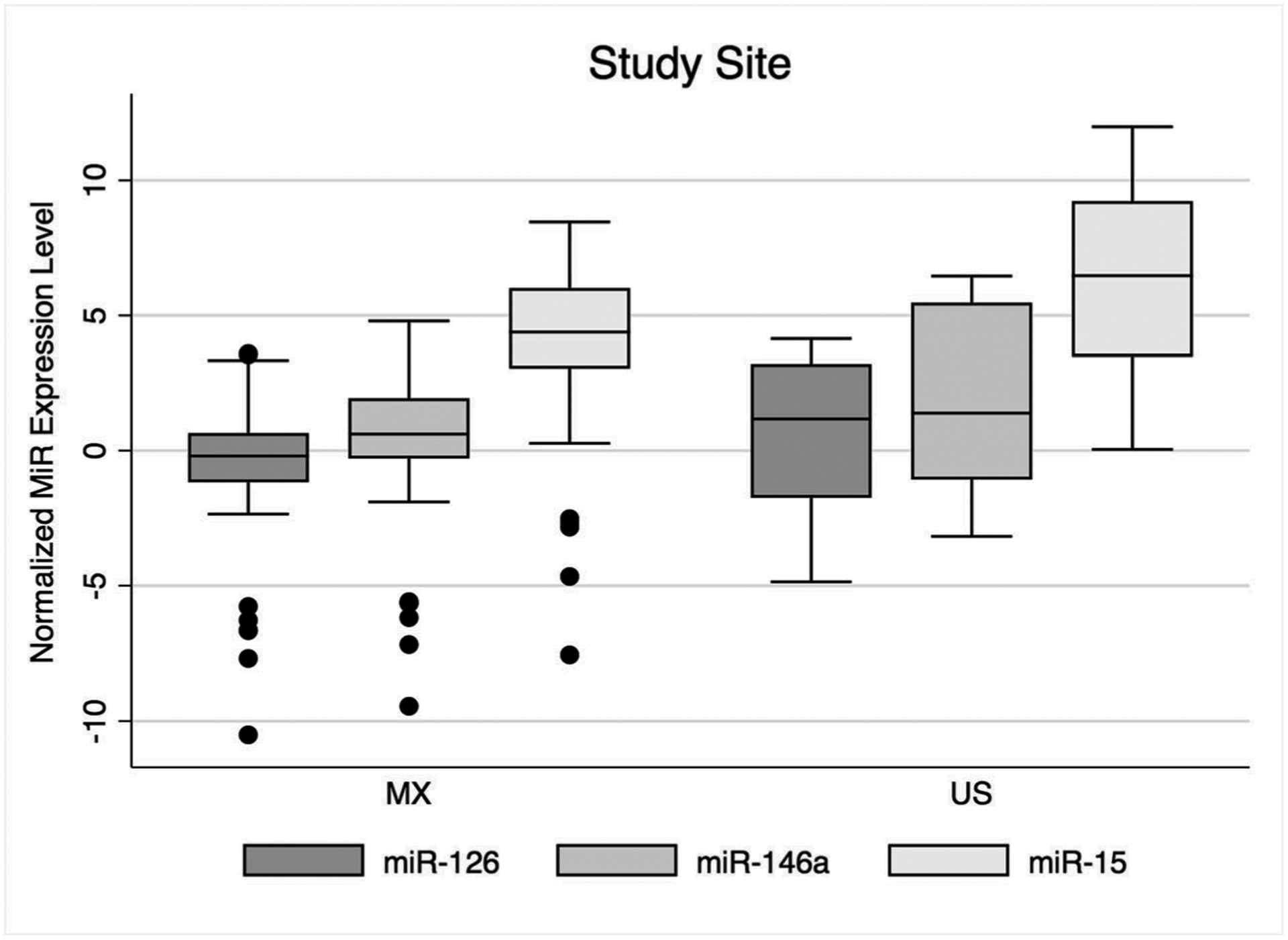
Distribution of normalized microRNA expression levels by study site. Box and whisker plots show maximum (upper horizontal line), 75th percentile (upper border of box), 50th percentile/median (mid-line of box), 25th percentile (lower border of box), and minimum (lower horizontal line). Black dots represent outliers. MiR, microRNA; MX, Mexico; US, United States

The distribution of normalized (i.e., ΔCt) expression for each microRNA by study site is shown in Fig. 1. In unadjusted logistic regression models, both miR-146a (OR 0.83 (95% CI 0.67, 0.99)) and miR-15 (OR 0.79 (0.65, 0.97)) were significantly decreased in individuals from the Mexico study site compared to the US site (Table 4). In a model adjusted for age and BMI, miR-15 remained significantly lower in individuals from Mexico compared to the US (OR 0.76, (95% CI 0.60, 0.97)). When we further added hemoglobin A1c to the model, which was higher in participants from the US compared to Mexico, miR-15 was no longer significant. There was no interaction between miR-15 and hemoglobin A1c. There was a significant interaction between miR-146a and BMI in both unadjusted and age- and BMI-adjusted linear regression models for fasting blood glucose.

**Fig. 2:**
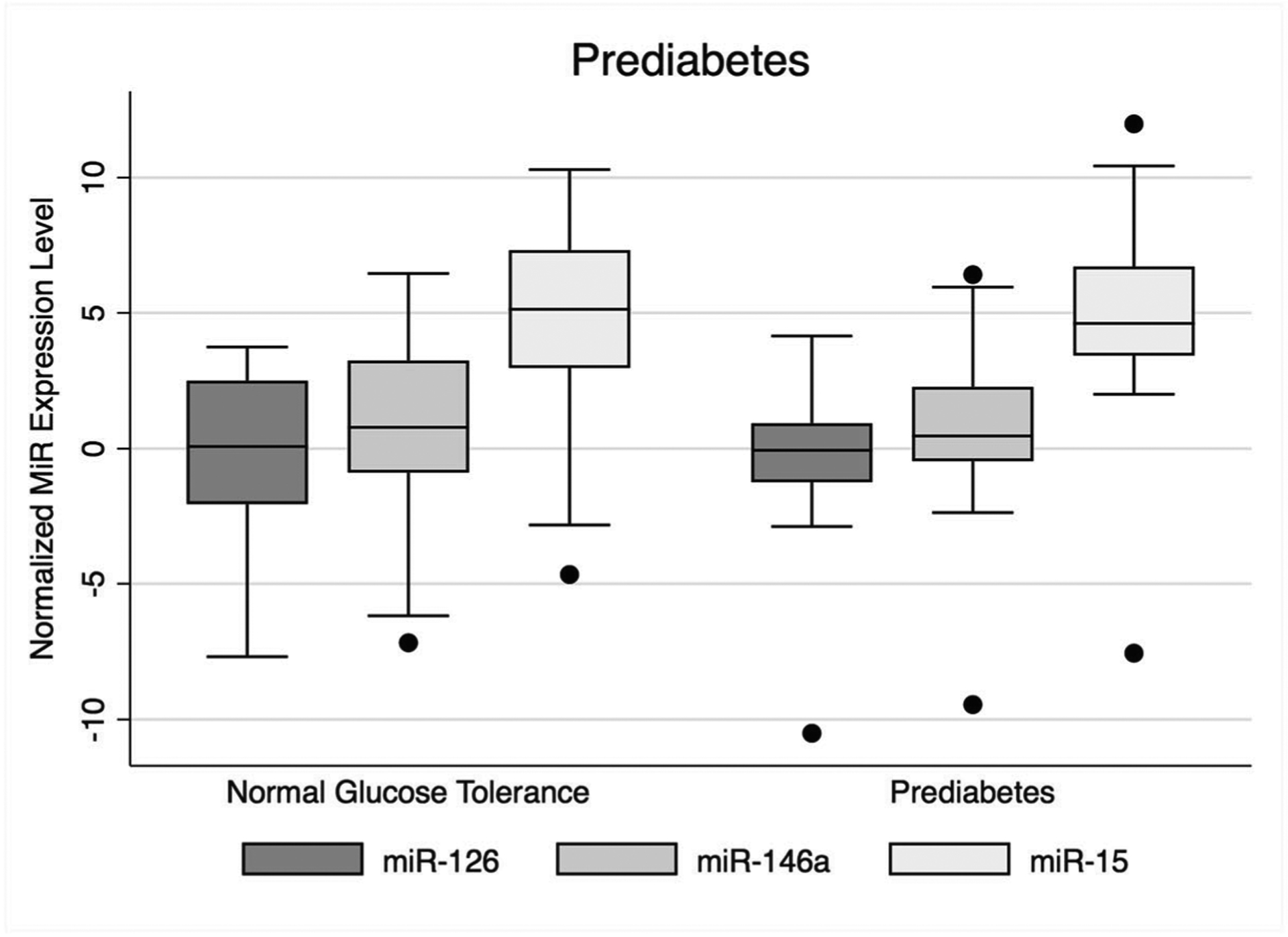
Distribution of normalized microRNA expression levels by glycemic status. Box and whisker plots show 95th percentile (upper horizontal line), 75th percentile (upper border of box), 50th percentile/median (mid-line of box), 25th percentile (lower border of box), and minimum (lower horizontal line). Black dots represent outliers. MiR, microRNA; MX, Mexico; US, United States

The distribution of normalized (i.e., ΔCt) expression for each microRNA by glycemic status is shown in Fig. 2. In unadjusted and sex- and BMI-adjusted logistic regression models, no microRNAs were significantly associated with higher odds for prediabetes. In unadjusted and sex- and BMI-adjusted linear regression models, no microRNAs were significantly associated with fasting blood glucose. However, there was a significant interaction between miR-146a and BMI in a linear regression model for fasting blood glucose (*β* = − 0.16, 95% CI (− 0.32, − 0.01)). The test for interaction was not significant for miR-126 and miR-15.

## Discussion

Compared to prior studies focused on relationships between circulating microRNAs and risk for type 2 diabetes, we did not find significant associations between miR-126, miR-146a, or miR-15 and prediabetes. Prior studies were primarily conducted in European or Asian populations [12, 23, 24], whereas we studied Latinos. We did identify a significant association between miR-146a and BMI, which is a relationship that has previously been observed in several studies of Europeans [25]. We also identified differences in expression levels of miR-146a and miR-15 between individuals living in or near Leon, Mexico, compared with individuals living in the US who self-identified as Latino.

MiR-146a has previously been associated with risk for type 2 diabetes in numerous studies, including a meta-analysis [24, 26]. Inflammation is one of the potential mechanisms by which miR-146a is hypothesized to have an effect on risk for type 2 diabetes and related conditions [27, 28]. There is strong experimental evidence that biological pathways from the Kyoto Encyclopedia of Genes and Genomes (KEGG) are targeted by miR-146, including ones related to inflammation (e.g., nuclear factor-κβ (NF-κβ) signaling pathway, Toll-like receptor signaling pathway, tumor necrosis factor-α signaling pathway) [29]. Findings from prior studies have been inconsistent in terms of the direction of the association (i.e., increased versus decreased expression of miR-146a and increased risk for type 2 diabetes). These discrepancies may partly result from differences in the tissue source from which microRNAs were obtained (e.g., plasma versus peripheral blood mononuclear cells (PBMCs)) and cross-sectional study design, which does not allow for characterization of where an individual lies on the glycemic spectrum. Our study did not find any association between miR-146a and prediabetes, which may be attributed in part due to differences in study design, including the examples listed above [26]. Another possible explanation for our null findings is that prior studies were primarily focused on European and Asian populations [12]. One prior study of microRNAs and risk for type 2 diabetes included Mexicans [30]. MiR-146a was significantly decreased in individuals with type 2 diabetes compared to healthy controls and was significantly correlated with BMI [30]; however, microRNAs were obtained from PBMCs, whereas our study focused on circulating microRNAs from plasma. Another study identified decreased expression of miR-146a associated with type 2 diabetes in Ecuadoreans and correlations between miR-146a and inflammatory markers [31]. Both of these prior studies were cross-sectional and included individuals with a diagnosis of type 2 diabetes, whereas our study focused on prediabetes. There are many physiological changes across the glycemic spectrum from normal glucose tolerance to impaired fasting glucose (i.e., prediabetes) to type 2 diabetes that are not captured in cross-sectional study designs that use only fasting blood glucose to assess glycemic status.

Overweight and obesity are major risk factors for type 2 diabetes, with approximately 40–70% of individuals at high risk for type 2 diabetes being overweight or obese [32]. We observed a significant correlation between miR-146a and BMI and a significant interaction between these two variables in determining odds for prediabetes versus normal glucose tolerance. MiR-146a has previously been associated with obesity in animal models [33, 34] and obesity-related inflammation in human adipocytes [35]. In human studies, miR-146a was increased in obese Chinese children and Chinese adults with type 2 diabetes and in functional in vitro and animal model studies increased miR-146a impaired β-cell function and insulin secretion [23]. Another KEGG pathway targeted by miR-146a is the adipocytokine signaling pathway. Overweight and obesity cause inflammation in part through the activity of adipocytokines, which are inflammatory molecules generated in adipose tissue [36–38]. The significant interaction that we observed between miR-146 and BMI suggests that the effect of BMI on prediabetes depends on the expression level of miR-146a, or vice versa. The relationship between miR-146a and risk for type 2 diabetes may be linked to its effect on body composition and/or obesity-related inflammation. Future studies that include a longitudinal design, gold-standard assessments of body composition and glycemic status, and functional analysis of the impact of miR-146a on genes related to inflammation and obesity may further shed light on these relationships.

Prior studies identified associations between miR-15 and risk for type 2 diabetes [39, 40]. Baseline levels of miR-15 were lower in Spanish individuals who developed type 2 diabetes after 5 years, though miR-15 was not significantly associated with other measures of risk for type 2 diabetes (i.e., fasting blood glucose, hemoglobin A1c, measures of insulin sensitivity) [40]. A study of African-Americans identified a U-shaped curve in the relationship between miR-15 and the glycemic trajectory, with lower expression of miR-15 in individuals with prediabetes compared to individuals with normal fasting glucose or type 2 diabetes [39]. In the group with type 2 diabetes, miR-15 was associated with body weight and body mass index, but not hemoglobin A1c, and none of these associations was observed in the group with prediabetes [39]. In addition, miR-15 was able to discriminate between type 2 diabetes and prediabetes and between prediabetes and normal blood glucose, although these models were not compared to any other predictive or discriminatory models [39]. Our study showed that hemoglobin A1c, which differed between study sites (i.e., US versus Mexico) attenuated the relationship between miR-15 and study site. The relationship between miR-15 and hemoglobin A1c remains relatively unstudied. Mechanistic studies of miR-15 identified regulation of NF-κβ [41, 42] with corresponding increases in the inflammatory interleukin-8 and interferon-γ markers [42], suggesting that miR-15 may also contribute to regulation of inflammation observed in individuals at risk for type 2 diabetes.

MiR-126 was previously associated with risk for type 2 diabetes in numerous studies [14, 43]. Insights from mechanistic studies of miR-126 showed that this microRNA is associated with endothelial cell function [44–46], and therefore, differential expression may be associated with consequences from type 2 diabetes and elevated blood glucose levels [47]. Prior studies that identified associations between miR-126 and risk for type 2 diabetes focused on primarily European and Asian racial groups [43, 48–51]. Very little has been reported about microRNAs associated with risk for type 2 diabetes in Latino populations. Further studies are needed to validate our finding that miR-126 may not be associated with prediabetes in Latinos.

The Latino racial group category includes individuals from vast geographic regions with highly admixed genetic characteristics [6]. Characterization of individuals by this broad criterion may lack specificity about the degree of genetic similarity. For example, Latinos living in California have different genetic characteristics compared with Latinos living on the East Coast of the US or in Mexico and Central and South America [52]. Prior studies that included individuals of Latino origin (e.g., Ecuadorean) may have been genetically dissimilar to our study sample. In order to accurately assess genetic similarity between individuals from the Latino racial group, genetic admixture analysis is needed. Furthermore, behavioral and lifestyle factors that impact risk for type 2 diabetes vary considerably between individuals who are broadly categorized as Latino. Inconsistencies in the associations between individual microRNAs and risk factors for type 2 diabetes may be the result of not only differences between racial groups in terms of genetic admixture/genetic risk and behavioral/lifestyle factors but also differences within racial groups (i.e., Latinos) that are unaccounted for by this very general categorization.

A limitation of this study and the majority of studies to date is the cross-sectional design. Development of type 2 diabetes occurs on a continuum, and cross-sectional studies fail to identify where on this continuum an individual may fall. Even within the clinically assigned categories of normal glucose tolerance, prediabetes, and type 2 diabetes, there may be differences in the underlying pathophysiology that impact expression levels of circulating microRNAs. Clinical and molecular data collection for this study was carried out at separate study sites. However, the laboratory protocols were identical at both sites, and study personnel were trained on the molecular data collection protocols at the US site. All data analysis was performed at a single site (US). There were some differences in demographic and clinical characteristics between the study sites, which were included as covariates in models. The genetic ancestry of all participants is not known, and the degree of genetic similarity between individuals is not known. Environmental and lifestyle factors were not assessed and conclusions about the impact of these potential risk factors on the associations between individual microRNAs and prediabetes cannot be made.

Circulating microRNAs are emerging as promising biomarkers for risk for type 2 diabetes. However, the majority of studies to date have primarily included individuals who identify as non-Hispanic white/European or Asian. Latinos have a particularly high prevalence of type 2 diabetes, and the reasons for this are not well understood. Given that circulating microRNAs capture the combined impact of genetic predisposition with responses to environmental and lifestyle factors, they may provide new insights about the reasons for increased risk for type 2 diabetes in some individuals and populations.

## Figures and Tables

**Table 1:** Demographic and clinical characteristics by study site

% (n) or average ± standard deviation	US Latinos (*n* = 21)	Mexican (*n* = 45)	*p* value
Age	51 ± 7	46 ± 8	< 0.05
Sex (male)	24 (5)	31 (14)	0.54
Body mass index (kg/m^2^)	35.9 ± 8.1	29.8 ± 3.8	< 0.05
Weight (kg)	92.3 ± 18.2	81.8 ± 11.4	< 0.05
Fasting blood glucose (mg/dL)	101 ± 11	99 ± 14	0.44
Hemoglobin A1c (%)	6.0 ± 0.3	4.4 ± 0.5	< 0.001
Prediabetes (%)	57 (12)	67 (24)	0.77
Total cholesterol (mg/dL)	216 ± 44	194 ± 34	0.06
LDL-c (mg/dL)	134 ± 37	120 ± 30	0.13
HDL-c (mg/dL)	47 ± 10	39 ± 11	<0.05
Triglycerides (mg/dL)	176 ± 68	165 ± 6	0.58

**Table 2:** Demographic and clinical characteristics by glycemic status

	Normal Glucose Tolerance (*n* = 30)	Prediabetes (*n* = 36)	*p* value
Study site (Mexico)	70 (21)	67 (24)	0.77
Age (years)	47 ±9	48 ±8	0.40
Sex (male)	43 (13)	17 (6)	< 0.05
Body mass index (kg/m^2^)	29.7 ± 5.8	34.4 ± 6.0	< 0.05
Weight (kg)	81.2 ± 13.6	88.6 ± 14.5	0.05
Fasting blood glucose (mg/dL)	89 ± 8	109 ± 8	< 0.001
Hemoglobin A1c (%)	4.8 ± 0.7	5.1 ± 0.9	0.18
Total cholesterol (mg/dL)	201 ± 35	201 ± 41	0.96
LDL-c (mg/dL)	125 ± 27	123 ± 37	0.85
HDL-c (mg/dL)	41 ± 9	42 ± 13	0.67
Triglycerides (mg/dL)	170 ± 82	167 ± 77	0.86

**Table 3:** Correlation coefficients between individual microRNAs and covariates

	MiR-126	MiR-146a	MiR-15	Age	Sex	BMI	FBG
MiR-126	1.0000						
MiR-146a	*0.9538*	1.0000					
	*< 0.001*						
MiR-15	*0.7406*	*0.6792*	1.0000				
	*< 0.001*	*< 0.001*					
Age	− 0.1470	− 0.0997	− 0.0646	1.0000			
	0.2388	0.4258	0.6063				
Sex	− 0.2197	− 0.1761	− 0.1401	0.3649	1.0000		
	0.0763	0.1573	0.2620	0.0026			
BMI	0.1687	*0.2815*	0.0362	*0.3245*	0.2314	1.0000	
	0.1757	*0.0220*	0.7730	*0.0078*	0.0616		
FBG	0.0971	0.1235	0.1928	0.0917	0.1446	*0.3144*	1.0000
	0.4379	0.3232	0.1208	0.4639	0.2466	*0.0101*	

Italicized font indicates values that were statistically significant

*BMI*, body mass index; *FBG*, fasting blood glucose

**Table 4:** Odds ratios for prediabetes and study site

	miR-126, OR (95% CI)	miR-146a, OR (95% CI)	miR-15, OR (95% CI)
Prediabetes			
Unadjusted	1.03 (0.88, 1.22)	1.02 (0.89, 1.19)	1.05 (0.92, 1.21)
Adjusted*	1.05 (0.87, 1.27)	1.02 (0.85, 1.22)	1.09 (0.93, 1.27)
Study site^#^			
Unadjusted	0.86 (0.70, 1.05)	*0.83 (0.67, 0.99)*	*0.79 (0.65, 0.97)*
Adjusted^$^	0.99 (0.71, 1.14)	0.87 (0.69, 1.10)	*0.76 (0.60, 0.97)*

Italicized font indicates values that were statistically significant

* Models are adjusted for sex and BMI

# United States is the reference site

$ Models are adjusted for age and BMI

*BMI*, body mass index; *CI* confidence interval; *OR*, odds ratio
